# Machine learning-based radiomic analysis and growth visualization for ablation site recurrence diagnosis in follow-up CT

**DOI:** 10.1007/s00261-023-04178-4

**Published:** 2024-01-30

**Authors:** Yunchao Yin, Robbert J. de Haas, Natalia Alves, Jan Pieter Pennings, Simeon J. S. Ruiter, Thomas C. Kwee, Derya Yakar

**Affiliations:** 1 Department of Radiology, Medical Imaging Center Groningen, University of Groningen, University Medical Center Groningen, PO Box 30001, 9700 RB Groningen, The Netherlands; 2 Diagnostic Image Analysis Group, Department of Medical Imaging, Radboud University Medical Center, 6500 HB Nijmegen, The Netherlands; 3 Department of Hepatobiliary Surgery and Liver Transplantation, University of Groningen, University Medical Center Groningen, PO Box 30001, 9700 RB Groningen, The Netherlands; 4 Department of Radiology, Netherlands Cancer Institute, Amsterdam, The Netherlands

**Keywords:** Radiofrequency ablation, Recurrence, Machine learning, Hepatocellular carcinoma, Neoplasm metastasis

## Abstract

**Objectives:**

Detecting ablation site recurrence (ASR) after thermal ablation remains a challenge for radiologists due to the similarity between tumor recurrence and post-ablative changes. Radiomic analysis and machine learning methods may show additional value in addressing this challenge. The present study primarily sought to determine the efficacy of radiomic analysis in detecting ASR on follow-up computed tomography (CT) scans. The second aim was to develop a visualization tool capable of emphasizing regions of ASR between follow-up scans in individual patients.

**Materials and methods:**

Lasso regression and Extreme Gradient Boosting (XGBoost) classifiers were employed for modeling radiomic features extracted from regions of interest delineated by two radiologists. A leave-one-out test (LOOT) was utilized for performance evaluation. A visualization method, creating difference heatmaps (diff-maps) between two follow-up scans, was developed to emphasize regions of growth and thereby highlighting potential ASR.

**Results:**

A total of 55 patients, including 20 with and 35 without ASR, were included in the radiomic analysis. The best performing model was achieved by Lasso regression tested with the LOOT approach, reaching an area under the curve (AUC) of 0.97 and an accuracy of 92.73%. The XGBoost classifier demonstrated better performance when trained with all extracted radiomic features than without feature selection, achieving an AUC of 0.93 and an accuracy of 89.09%. The diff-maps correctly highlighted post-ablative liver tumor recurrence in all patients.

**Conclusions:**

Machine learning-based radiomic analysis and growth visualization proved effective in detecting ablation site recurrence on follow-up CT scans.

**Supplementary Information:**

The online version contains supplementary material available at 10.1007/s00261-023-04178-4.

## Introduction

Hepatocellular carcinoma (HCC) represents one of the most frequently occurring malignancies worldwide, exhibiting a 5-year survival rate of approximately 18%. In 2020, nearly 906,000 individuals were diagnosed with liver cancer, with HCC being the most prevalent form [1, 2]. Colorectal cancer ranks among the top three most prevalent cancers globally. Approximately 50% of patients with colorectal cancer eventually develop liver metastases, which pose a significant challenge following curative treatment for colorectal cancer and contribute to the overall mortality rate [3–5]. Curative interventions for HCC encompass surgical resection and liver transplantation. Nonetheless, numerous patients are ineligible for such treatments due to for example multifocal disease, metastases, inadequate hepatic reserve, and organ donor scarcity. Similar principles hold true for colorectal liver metastases, which are not always amenable for surgical resection. As a result, thermal ablation (TA) has emerged as a widely employed minimally invasive treatment modality with promising local tumor control rates and long-term outcomes [6–8].

The main drawback of TA is the relatively frequent occurrence of viable tumor at the edge of the ablation zone which is called ablation site recurrence (ASR). Risk factors for ASR include insufficient ablative margins, tumor location, and tumor morphology [9–12]. Accurate and timely diagnosis of ASR is crucial to ensure the option of (minimally invasive) re-treatments with curative intent leading to the best long-term outcomes [13]. However, diagnosing ASR on follow-up computed tomography (CT) scans remains difficult, even for experienced radiologists due to the similarity between post-ablative necrosis/perilesional inflammation and true ASR [14–16].

Radiomics, an emerging methodology in quantitative medical image analysis, encompasses the extraction of an extensive array of hand-crafted radiomic features from medical images. These features translate visual information and phenotypic traits into numerical and quantitative data amenable to machine learning algorithm modeling and analysis [17–19]. Because of these capabilities, radiomic analyses have demonstrated potential in enhancing clinical outcomes [20, 21].

The present study primarily sought to determine the efficacy of radiomic analysis in detecting ablation site recurrence on follow-up CT scans after thermal ablation of malignant liver tumors. The secondary aim was to develop a visualization tool capable of emphasizing regions of recurrence between follow-up scans in individual patients.

## Material and methods

### Study design and patient selection

A retrospective cohort of adult patients who underwent TA for liver tumors including HCC and metastases from colorectal and breast cancer between 2008 and 2020 was established from the electronic patient records at the XXX. At our center, the follow-up protocol after TA consists of a first CT scan one week after TA, followed by CT scans every 4 months during the first two years, and thereafter every six months up to five years after the treatment.

All reports of follow-up CT scans after TA, generated by abdominal radiologist as part of routine patient care, were retrospectively scrutinized for the evidence of recurrent disease. ASR was characterized by the emergence of a contrast-enhancing lesion either within or in the immediate vicinity of the ablation zone. Concurrently, the largest diameter of these lesions maintains direct contact with the ablation zone [22]. In case of radiological evidence of ASR with histopathological confirmation, patients were classified as the positive patient group. In this ‘ASR-positive’ patient group, the follow-up CT scans on which the ASR was identified were used for the radiomic analysis (average 12 months [interquartile range: 5–17 months] after the date of TA).

A control group was established by randomly selecting patients until 2020 from the cohort with follow-up CT scans without evidence of ASR. In these patients, the most recent follow-up CT scan was used (average 18 months [interquartile range: 12–23 months] after the TA date) for radiomic analysis.

**Fig. 1:**
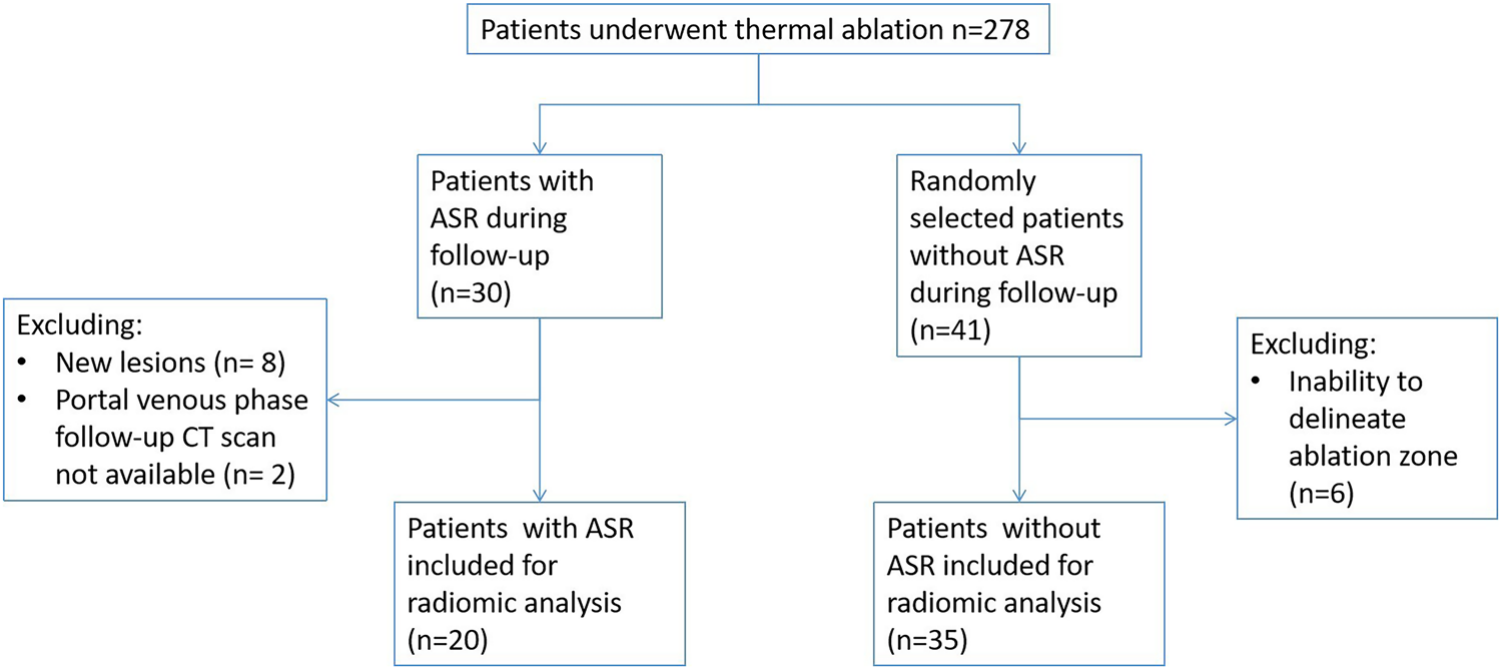
Patient exclusion diagram

Exclusion criteria (Fig. 1) for radiomic analysis were (1) unavailability of contrast-enhanced portal venous phase CT scans, such as cases where ASR was confirmed through follow-up magnetic resonance (MR) or positron emission tomography (PET) scans, or when only the arterial phase was accessible in the picture archiving system; (2) distant intrahepatic liver lesions, identified by a radiologist as a novel lesion rather than ASR; (3) inability to delineate the ablation zone on the latest follow-up scan in patients in the control group. This inability arises when the ablation zone is overgrown by normal liver tissue, rendering it invisible in the patient's most recent follow-up scan. This indicates normalization and, consequently, the absence of recurrence.

Multivendor CT systems were employed, with scan parameters harmonized between our hospital and referring institutions as follows: automatic tube current modulation and tube voltage selection, 1 mm slice thickness, 75-s delay following the intravenous injection of 90–100 mL contrast medium at a 3.6–4.0 mL/s flow rate, succeeded by a 32 mL saline solution. The Institutional Review Board granted approval, and the requirement for written informed consent was waived.

### Region of interest and image processing

**Fig. 2:**
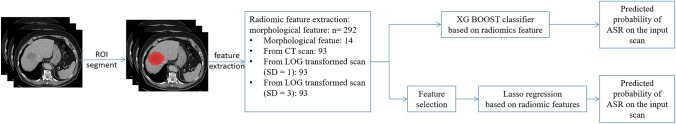
Workflow of radiomic analysis

**Fig. 3:**
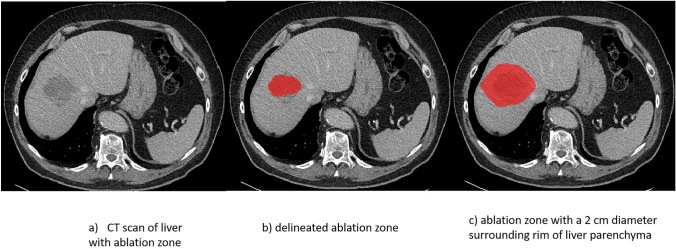
Example of Region of Interest

The entire workflow of the study is demonstrated in Fig. 2. The ablation zone and a 2 cm diameter surrounding rim of liver parenchyma constituted the region of interest (ROI). Ablation zones were delineated by two experienced abdominal radiologists separately on different parts of the dataset, with the mask of the surrounding liver parenchyma rim being automatically generated through morphological dilation of the delineated ablation zone. Figure 3 shows examples of binary masks for the ablation zone and adjacent liver parenchyma rim.

To modulate contrast and brightness of the CT scan, thereby augmenting soft tissue visibility, a soft tissue window centered at 50 HU with a width of 400 HU was implemented. For normalization, all images employed in the radiomic analysis were resampled to identical spacing [1.0 mm, 1.0 mm, 2.0 mm] using a B-spline interpolator. Gray-level discretization employed a fixed bin size method and tested the size set of {5, 15, 25}.

### Radiomic features

Radiomic features represent a collection of quantitative measurements derived from medical images, translating radiological visual information into numerical data. A predefined set of radiomic features according to the Image Biomarker Standardization Initiative (IBSI) was extracted from the original pre-processed CT scans [23], encompassing morphological features, first-order statistical features, gray level co-occurrence matrix features, gray level size zone matrix features, gray level run length matrix features, neighboring gray tone difference matrix features, and gray level dependence matrix features. Additionally, first-order statistical features and texture features were also extracted from Laplacian of Gaussian (LoG) filter-transformed CT scans, since the LoG filter enhances the visibility of subtle image structures, such as edges. The amalgamation of radiomic features from the original and LoG-transformed scans provides more comprehensive insight into underlying tissue characteristics. Feature extraction was executed using Python 3.7.9 in the open-source library Pyradiomics 3.0 [24].

Extracted radiomic features may exhibit strong linear relationships with one another. To address collinearity, Pearson correlation coefficients between radiomic features were computed. Radiomic feature groups exhibiting Pearson correlation coefficients > 0.8 were deemed highly correlated and therefore removed to decrease dataset dimensionality and mitigate collinearity issues.

### Machine learning classifiers

Owing to the limited dataset size, logistic regression with L1 penalty (Lasso regression) was employed for feature modeling, as it is apt for small-scale data analysis tasks [25]. The L1 penalty served as the regularization for the logistic regression classifier, penalizing high-valued regression coefficients to eliminate redundant features and reduce multicollinearity in feature sets. The classifier automatically selected radiomic features related to training targets during training. Feature importance for Lasso regression was gauged by the corresponding feature weights in trained classifiers. Furthermore, extreme gradient boosting (XGBoost) methods were also utilized for radiomic feature modeling. XGBoost classifiers, constructed by decision trees, facilitate powerful feature selection to distinguish ASR at each split node [26]. Feature importance for the XGBoost classifier was measured by Gini importance (mean decrease in impurity) [27].

To furnish an unbiased performance estimate of trained classifiers, a leave-one-out test (LOOT) approach was employed. In LOOT, the dataset was divided into n subsets, where n represents the number of patients in the entire dataset. For each subset, a model was trained using n-1 samples based on five-fold cross-validation. The trained model was subsequently tested on the held-out sample to evaluate performance. This process was repeated n times, with results computed based on predictions of held-out samples in each subset. Additionally, the imbalanced dataset, with more patients lacking ASR than those with ASR could influence machine learning model performance [28, 29]. Therefore, a class weight of 1.5 was applied to the minority class. By increasing the weight of patients with ASR, the classifier was compelled to consider the asymmetry of cost error between the positive and control groups. The model would incur a greater penalty for misclassifying ASR patients during training. The model was developed using the open-source library scikit-learn 0.23.2 with Python 3.7.9 [30].

### Visualization method for post-ablative grown region

Diagnosing ASR can be challenging for radiologists due to subtle tumor size and the similarity between ASR and post-ablative necrosis and perilesional inflammation. It was hypothesized that malignant recurrent tumors exhibit growth between two follow-up scans; thus, emphasizing the differences between follow-up scans could potentially assist radiologists in focusing on the grown region of disease recurrence, making it more accessible to visualize and identify ASR.

To generate a heatmap (diff-map) highlighting differences between two follow-up scans, the images were aligned using elastix software [31]. Liver segmentation on the CT ensured accurate registration across scans. Subsequently, the diff-map was generated by subtracting the two registered follow-up scans. To further refine the diff-map, it was smoothed using a Gaussian kernel with a standard deviation of 2.5, and then normalized to a range of 0 to 1. Regions exhibiting growth during the time interval between the two follow-up scans were characterized by larger differences in gray values on the scans, thus emphasizing the disease recurrence regions on the diff-map.

## Results

### Study population characteristics

In our center, 278 patients underwent thermal ablation for malignant liver tumors. Of these, 30 (10.8%) were identified with ASR. During patient selection, 2 patients were excluded due to the absence of follow-up CT scans on the date of ASR diagnosis, 8 patients were excluded because their liver tumors on follow-up CT were considered as new lesions.

For the control group, 41 patients without ASR were randomly selected. Six of these were excluded as their ablation zones could not be delineated on the CT scan.. Finally, 20 patients with ASR and 35 without ASR were eligible for radiomic analysis (Fig. 1).

The median age of the cohort was 67 years (interquartile range: 62–72 years), including 22 women (40%) and 33 men (60%). Among the liver lesions included in the analysis, 18 (32.73%) were HCC, 36 (65.45%) were colorectal metastases, and 1 (1.82%) concerned breast metastasis. Further patient characteristics can be found in Table 1.

**Table 1:** Patient demographics

Characteristic	Positive ASR	Negative ASR	P value	Total number	
Median age, IQR	68 (62–73)	67 (62–72)	69 (61–74)	P < 0.05	55 (100%)
Gender	Female	7 (12.73%)	15 (27.27%)	P < 0.05	22 (40%)
	Male	13 (23.64%)	20 (36.36%)		33 (60%)
Tumor type	HCC	7 (12.73%)	11 (20.00%)	N/A	18 (32.73%)
	Colon metastasis	8 (14.55%)	12 (21.82%)		20(36.36%)
	Rectal metastasis	1 (1.82%)	8 (14.55%)		9(16.36%)
	Breast metastasis	1 (1.82%)	0 (0.00%)		1(1.82%)
	Unknown metastasis	3 (5.45%)	4 (7.27%)		7(12.73%)
Cirrhosis	Absence	14 (25.45%)	27 (49.09%)	P < 0.05	41 (74.55%)
	Presence	6 (10.91)	8 (14.55%)		14 (25.45%)
Etiological cause	HBV	1 (1.82%)	2 (3.64%)	N/A	3 (5.45%)
	HCV	3 (5.45%)	0 (0.00%)		3 (5.45%)
	Alcohol	4 (7.27%)	5 (9.09%)		9 (16.36%)
	Auto-immune	0 (0.00%)	0 (0.00%)		0 (0.00%)
	Wilson	0 (0.00%)	0 (0.00%)		0 (0.00%)
	Biliaire atresie	1 (1.82%)	0 (0.00%)		1 (1.82%)
	Steatohepatitis	3 (5.45%)	1 (1.82%)		4 (7.27%)
	Primair scleroserende cholangitis	1 (1.82%)	1 (1.82%)		2 (3.64%)
	Unknown	7 (12.73%)	26 (47.27%)		33 (60.00%)

*ASR* ablation site recurrence, *IQR* Interquartile Range, *HCC* hepatocellular carcinoma, *HBV* hepatitis B virus, *HCV* hepatitis c virus

### Radiomic model development

In this study, a total of 292 radiomic features were extracted from the ROI within the images. These features included 14 morphological features of the ROI, 93 features extracted from the ROI on the original processed CT scans, and 93 features extracted from the ROI on images transformed by LoG filters with standard deviations of 1 and 3, respectively. A selection of 95 radiomic features was made based on Pearson correlation coefficients for model training.

**Fig. 4:**
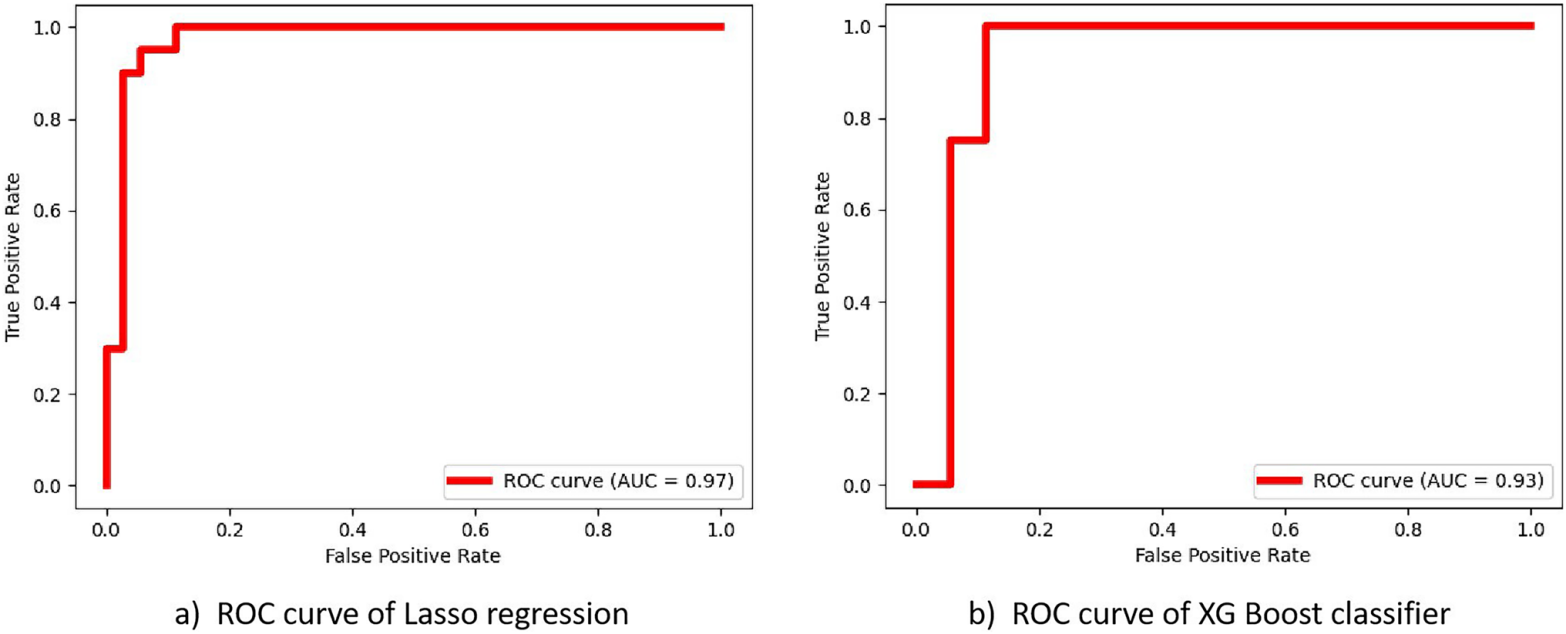
ROC curves for two different classifiers: **a** Lasso regression, which discriminates patients with and without post-ablative liver tumour recurrence; **b** XG Boost, which also discriminates patients with and without post-ablative liver tumour recurrence. *AUC* receiver operating characteristic curve

Table 2 lists the sensitivity, specificity, accuracy, and AUC of different classifiers, with corresponding ROC curves being illustrated in Fig. 4. The best-performing model was achieved using Lasso regression with a fixed bin size of 15, tested by the LOOT approach, yielding an AUC of 0.97 and an accuracy of 92.73%. Additionally, the XG Boosting classifier demonstrated improved performance when trained with all extracted radiomic features without feature selection, achieving an AUC of 0.93 and an accuracy of 89.09%. The performance of XG Boosting classifier without feature selection also slightly outperforms the classifier trained with feature selection on the training set with an accuracy of 98.18% and 96.36%, respectively. Table 3 lists the top five radiomic features selected based on weight ranking for each machine learning classifier. The complete radiomic feature list and corresponding weight rankings for each classifier can be found in Supplementary Tables I and II.

**Table 2:** Results of the radiomic analysis

Classifier	Accuracy (%) (95% CI)	Sensitivity (%) (95% CI)	Specificity (%) (95% CI)	AUC (95% CI)
Lasso regression	92.73 (90.90, 95.45)	95.00 (92.31, 1.0)	91.43 (88.46, 96.43)	0.97 (0.96,1.0)
XG Boost classifier	89.09 (81.82,90.91)	90.00 (73.33,92.31)	88.57 (84.62,93.33)	0.93 (0.90,0.98)

*AUC* area under the receiver operating characteristic curve, *CI* confidence interval

**Table 3:** The top 5 important features for radiomic analysis

	Lasso regression		XG Boost classifier
	Positive	Negative	
1st	original_glcm_Idmn	original_firstorder_Entropy	log-sigma-1-mm-3D_glcm_SumEntropy
2nd	original_glrlm_GrayLevelNonUniformity	original_glcm_Imc1	original_glszm_LargeAreaEmphasis
3rd	original_firstorder_10Percentile	log-sigma-3-mm-3D_glcm_Correlation	log-sigma-1-mm-3D_firstorder_Uniformity
4th	original_shape_Maximum2DDiameterColumn	log-sigma-3-mm-3D_glcm_Autocorrelation	log-sigma-1-mm-3D_glcm_ClusterTendency
5th	original_firstorder_Uniformity	log-sigma-3-mm-3D_glszm_SizeZoneNonUniformityNormalized	original_firstorder_Entropy
Total number of weighted features	26	10	40

The full list of features and corresponding weights in each classifier can be found in the supplementary materials

### Visualization of Diff-Map

**Fig. 5:**
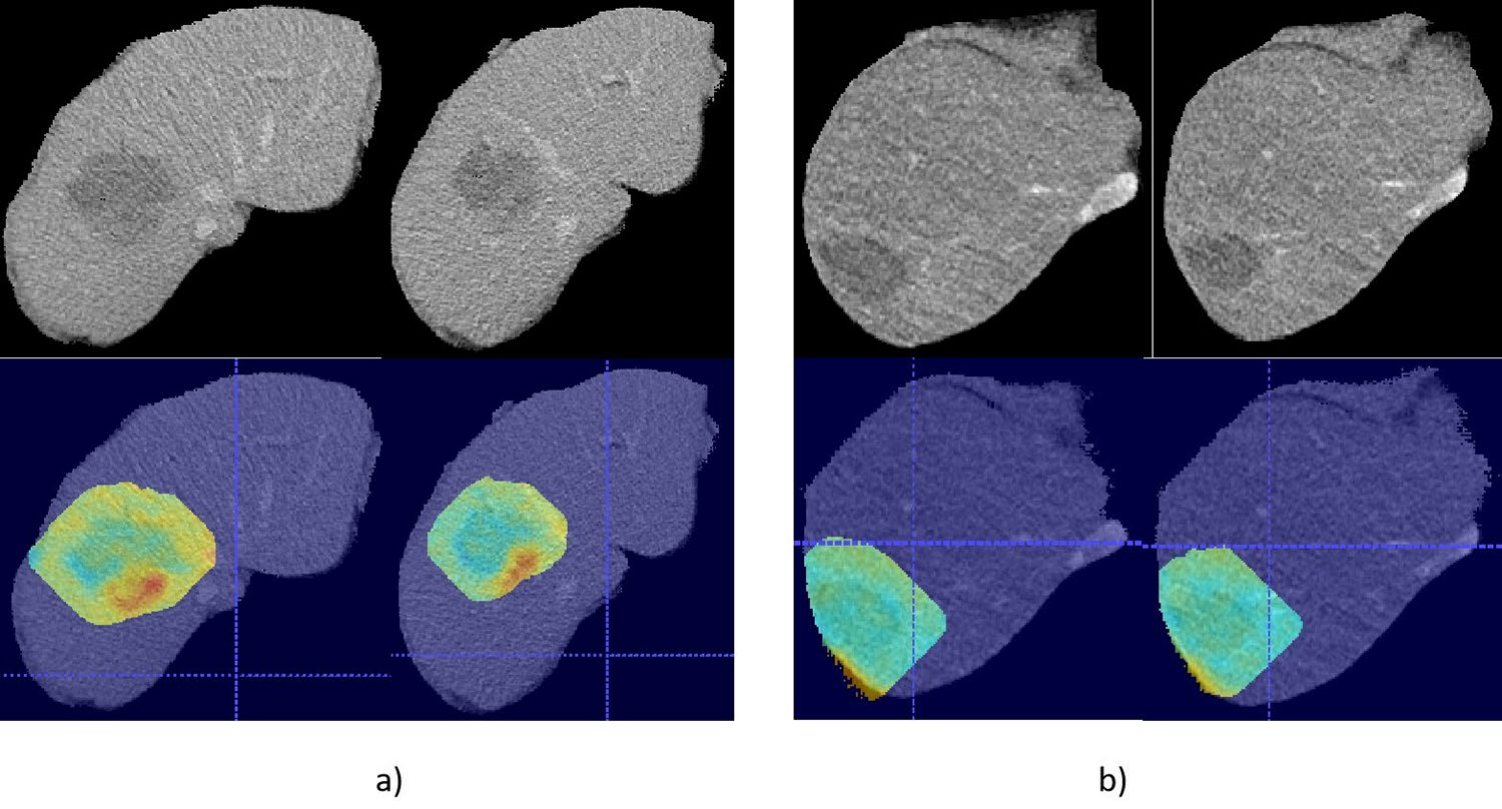
An example of diff-maps overlaid on a CT scan for two different scenarios: **a** The diff-maps for a patient with a post-ablative liver tumor recurrence, where the recurrent local tumor is highlighted with red color on the diff-maps. **b** The diff-maps for a patient without any post-ablative liver tumor recurrence, where no region is emphasized in red color on the diff-map

Diff-maps were generated to accentuate the differences between two follow-up CT scans. The scans utilized for radiomic analysis and the prior scans were chosen to create diff-maps and further examine whether ASR were highlighted on the diff-maps. Figure 5 presents several examples of the generated diff-maps. In the diff-map of patients with ASR, the recurrence was correctly emphasized in each case. Conversely, in patients without ASR, the diff-map did not highlight any regions.

## Discussion

The current study aimed to assess the capability of radiomic analysis in discerning ASR on follow-up CT scans. Additionally, we developed a visualization technique that emphasizes regions of lesion growth between follow-up scans across distinct time periods for individual patients and examined whether ASR could be accurately highlighted. A total of 55 patients were included in the radiomic analysis, of whom 20 had ASR and 35 did not. Using the LOOT approach, Lasso regression achieved the highest AUC of 0.97 and the best accuracy of 92.73%. The diff-maps generated by the visualization method accurately highlighted ASR.

As accurately identifying ASR at the edge of the ablation zone remains a challenge for radiological visual interpretation [14–16], radiomic-based machine learning methods offer a quantitative approach for evaluating ASR independently of radiologists' subjectivity. Our study demonstrates promising results and suggests that these methods may serve as supportive tools to reduce subjectivity by providing differential diagnosis suggestions or guidance on suspicious lesions. Furthermore, the diff-maps could assist radiologists in focusing on regions of growth in follow-up scans, thus facilitating the early diagnosis of liver tumor recurrence. The early and accurate diagnosis of disease recurrence plays a critical role in optimizing patient outcomes, as it enables timely use of minimally invasive treatments, such as re-ablation, thereby increasing long-term patient survival [6, 32, 33]. Timely intervention in the early stages of disease recurrence can help mitigate the spread of disease to other organs, further emphasizing the importance of prompt and accurate diagnosis [34–36].

In addition to our approach focusing on radiomic analysis for the detection of ASR, several studies have explored the diagnosis of liver tumor recurrence following curative treatments such as resection and transplantation based on radiomic analysis [41, 42]. Moreover, some research has focused on predicting the chances of recurrence based on pre-ablation scans, which could provide valuable insights for treatment planning and personalized therapeutic strategies [43, 44]. It is important to note the differences between our study and the aforementioned research. While our study aims to detect and visualize the ASR using follow-up CT scans, these other studies focus on recurrence diagnosis after resection or predicting the likelihood of recurrence before ablation. This highlights the versatility of radiomic analysis and machine learning techniques in addressing various aspects of liver tumor management. Although a growing body of evidence exists supporting the application of advanced analytical methods in the diagnosis and prediction of liver tumor recurrence, further research is warranted to compare the performance of these different approaches and explore potential synergies to enhance the overall effectiveness of liver tumor management strategies.

In the current study both HCC and liver metastasis cases were included, reflecting a more diverse patient population and a broader range of liver tumor types. This is in contrast with many previous studies, which have primarily focused on either HCC or liver metastasis alone [45, 46]. The promising results obtained in our study, with an AUC of 0.97 and an accuracy of 92.73%, demonstrate the effectiveness of our machine learning-based radiomic analysis and growth visualization approaches in detecting ASR, regardless of the tumor origin. This suggests that our method may have broader clinical applicability and could potentially contribute to improved patient outcomes in different liver tumor types.

In the current study, the performance of the XG Boost classifier was inferior to Lasso regression. One potential reason could be the limited size of the patient cohort. XG Boost classifiers are more complex than Lasso regression and are better suited for handling larger and more intricate datasets. However, the patient cohort in this study was relatively small, which could increase the risk of overfitting for XG Boost classifiers [47, 48].

Another limitation of our study was the discrepancy in follow-up durations between the ASR-positive group (average 12 months) and the ASR-negative group (average 18 months). This difference arose because the ASR-positive group required shorter follow-up intervals due to the clinical urgency in confirming and managing recurrence. Conversely, the ASR-negative group typically underwent longer follow-up periods as a standard part of routine care. To mitigate this limitation, future research could establish a control group with follow-up times comparable to those of the ASR-positive group or design a prospective study to reduce the selection bias inherent in retrospective studies.

Although the radiomic analysis in our study achieved an AUC of 0.97 and an accuracy of 92.73%, its desig as a single-center study with a limited patient cohort may have limited the generalizability of our results. To address this limitation, future studies should be designed as multi-center investigations to create a larger and more diverse patient cohort for radiomic analysis. Evaluating radiomic-based machine learning models with external data is also crucial for assessing the generalization ability of these models.

The generated diff-maps accurately highlighted ASR, which could help draw radiologists' attention. However, other regions exhibiting differences on follow-up scans could also be emphasized on the diff-maps, such as vessels. This study used contrast-enhanced CT scans in the portal venous phase, and the intensity of vessels could vary depending on the amount of contrast agent and the time-delay after intravenous injection. Future studies could design a deep learning model to automatically segment ASR on follow-up scans when a larger patient cohort is available to circumvent this potential problem. The accuracy of the diff-map is contingent upon the quality of registration. To guarantee precise liver alignment, we pre-segmented the liver before registration. However, imprecise registration sometimes led to the highlighting of the liver's edges on the diff-map. The robust of the registration method could be further investigated and validated with larger patient cohort.

In conclusion, this study presents a novel approach to detecting ablation site recurrence through the application of radiomic analysis on follow-up CT scans and the development of a visualization method highlighting regions of lesion growth between scans. Our results demonstrate the potential of radiomic-based machine learning models serving as a valuable supportive tool for radiologists in their clinical practice. Furthermore, the diff-map visualization method may assist radiologists in identifying ablation site recurrence more easily and timely by emphasizing areas of growth on follow-up scans.

### Supplementary Information

Below is the link to the electronic supplementary material.

Supplementary file1 (XLSX 10 kb)

Supplementary file2 (XLSX 9 kb)
